# How to Accommodate the Emotional Dimensions of Advance Care Planning Using Motivational Interviewing and Conditional Medical Orders

**DOI:** 10.3390/healthcare10112257

**Published:** 2022-11-11

**Authors:** Richard B. Stuart, George Birchfield, Stephen Thielke

**Affiliations:** 1 Department of Psychiatry and Behavioral Sciences Seattle, University of Washington, Edmonds, WA 98020, USA; 2 Swedish Hospital-Edmonds, Edmonds, WA 98026, USA; 3 Evergreen Health Hospice Care Center, Kirkland, WA 98034, USA

**Keywords:** advance care planning, motivational interviewing, conditional medical order, emotion, advance directive

## Abstract

Palliative care discussions offer a unique opportunity for helping patients choose end-of-life (EOL) treatments. These are among the most difficult decisions in later life, and protecting patients’ ability to make these choices is one of healthcare’s strongest ethical mandates. Yet, traditional approaches to advance care planning (ACP) have only been moderately successful in helping patients make decisions that lead to treatments concordant with their values. In particular, neglect of attention to the emotions that occur during consideration of the end of one’s life contributes to patients’ difficulty with engaging in the process and following through on decisions. To improve ACP outcomes, providers can address the patient’s emotional experiences, and can use motivational interviewing as a way attend to elicit them and incorporate them into care planning. Applying personalizing emotion-attuned protocols like Conditional Medical Orders (CMO) also promotes this end.

## 1. Introduction

Palliative care is offered to patients with serve infirmities that may or not be terminal. There is general agreement that: “The goal of palliative care is to relieve the suffering of patients and their families by the comprehensive assessment and treatment of physical, psychosocial, and spiritual symptoms experienced by patients” [1]. This often involves helping patients find comfort by being able to plan their present and future care by creating advance care plans (ACP). This planning requires good communication among providers, patients, and their families, and ideally through shared decision-making. Effective communication is so critical that the Palliative Care Implementation Project stipulated “Patients’ experience of feeling heard and understood” as its first measurement criteria [2]. One key to good communication is sensitivity to the emotions that the contemplation of illness and death invariably evoke. Unlike the brief contacts in many instances of specialty care, patents tend to have more protracted contact with palliative care providers which increases the possibility of meaningful connection. Unfortunately, this sensitivity is elusive if, as often the case, the connection between cognition and emotion is overlooked.

Descartes’ declaration “I think therefore I am” fueled the Enlightenment’s obsession with cognition, which has persisted until now. But had Descartes known about resent landmark research, he would have expressed a doubly conditional phenomenon: “I feel, therefore I think, therefore I am” [3]. Neuroscience research has demonstrated that all processing in the brain involves emotion as much as or more than rationality. For instance, neurotensin instantly triggers a good/bad reaction in the amygdala to all sensory input [4]. This explains the classic finding that an evaluative dimension is the largest contributor to the meaning of most words in many languages [5]. It also accounts for the automaticity through which individuals begin an action before awareness of the intention to do so [6]. Studies like these highlight the need to consider both cognition and affect in all efforts to understand and manage human behavior [7]. They also demonstrate that: “behavior is the result of the interaction between what we believe and what we feel. If we want to change behavior, it is necessary to change the underlying beliefs and feelings related to that behavior” [8].

These mental processes apply equally throughout life, including planning around death. Yet, in many efforts to help patients with advance care planning, the only discussion involves logical constructs: “clinicians tend to focus on diagnosis, therapy, and cure [so] the imminence of death is often not openly and timely acknowledged in patients with advancing chronic illness” [9]. In the process, clinicians generally neglect the wide range of emotions that many patients experience when they contemplate serious illness and death. These include, but are not limited to, feelings of anger, fear, regret, guilt, loneliness and/or emptiness due to a lack of meaning [10]. Not all the feelings are negative: there may even be relief for those seeking to end the ravages of a terminal illness.

Ignoring emotions like these can seriously undermine the effectiveness of the most well-intended ACP efforts [11]. Without consonant attention to the emotional experiences about the process and content, patients are less likely to complete the needed steps. If something “feels” wrong or unpleasant, even if all the facts line up, they or their families may refuse to participate, may make unrealistic demands, or may acquiesce to the provider’s advice initially only to later do something else.

In order to minimize the risk of these unwelcome emotional outcomes, we suggest ways for providers to manage their own and their patients’ emotions during shared decision-making. We also recommend use of more patient-friendly materials to facilitate the process.

## 2. Why Providers’ Emotions Matter

Through a process of limbic regulation and the activity of mirror neurons, also known as “mood contagion”, humans and many other animals, covertly experience reactions that are sensed by other creatures, including pain and sadness [12,13]. The resulting social sensitivity can be regarded as a “sixth sense” to others’ feelings. Throughout the animal world, being able to quickly know when another individual is a threat or an opportunity aids survival. Without your verbalizing it, you may have noticed that your pet often becomes more attentive when you are sick or unhappy and flees when it senses your anger. This is an example of how the pet’s limbic system picks up messages from yours. Although they may not be aware of it, providers’ emotions have a similar impact on their patients, who readily sense anxiety, respectful interest, indifference, or disdain. In addition, providers’ emotions color the selection, timing, and manner in which they present alternatives.

Because emotions and healthcare decisions are inextricably intertwined, it is important to attend to the emotional tone of the way both providers and patients participate in discussions [11]. Providers who are ambivalent about an option may present it in an untimely manner or not at all, and those who disapprove of an option are apt to convey their feelings through nonverbal cues and verbal qualification of their messages, such as “this is probably not a good idea, but I have to mention this option…” [14]. Patients who feel that they are discounted or not well understood are unlikely to attend to presentations even if the information they are given is accurate and detailed.

## 3. Integrating Emotional Awareness into Shared Decision Making

In light of the patients’ and providers’ emotions, helping patients make decisions about end-of-life care is a skill that can be learned and must continually be refreshed [15]. It helps to begin by understanding the Transtheoretical Model of the Stages of Change that identifies movement from precontemplation through contemplation to planning, action, and maintenance [16]. Using this framework, motivational interviewing is an incredibly powerful tool for eliciting the patient’s perspectives on change in an emotionally safe discussion. Along with personality factors like resilience and executive functioning, the patient’s level of motivation influences the nature and intensity of the way options are presented. Whether the patient is reluctant to act, which requires more time and effort, or is ready to act, which expedites the process, motivational interviewing integrates emotion with cognition in the process of shared decision-making. It offers a toolkit for helping patients make prudent decisions that align with their values and preferences [17].

Motivation is a state of readiness or eagerness to change [6]. This is an emotional as well as cognitive process. Promoting motivation (whether through motivational interviewing or other techniques) requires a collaborative interaction in which practitioners provide essential information, nonjudgmentally discover and acknowledge the patient’s emotions, values and priorities, and negotiate a plan of action through which the patient can achieve a desired goal. Even in the best of circumstances, it is challenging to build the motivation to change directions; it is far more difficult when illness and death hang in the balance. Helping patients make such major changes during ACP requires careful attention to process and content.

### 3.1. Articulate and Manage Your Own Feelings about Shared Decision Making and ACP

As a provider, it is essential to identify and manage your own emotional own state. If you are tired, irritated, frustrated, or preoccupied with any significant aspect of your life, you will likely have difficulty connecting emotionally your patients. The following steps can help.

1. Understand and learn how to control your feelings about shared decision-making so you can give your patients the attention they deserve and allow them to be in charge of their own decisions.
2. Before meeting each new patient, pause for a few deep breaths to clear your brain and calm your nerves.
3. Accept the importance of patient perspectives on different courses of action. Patients are very unlikely to follow through on any recommendation about which they have doubts.
4. Identify the ways you feel about the options you will present to patients, and work on limiting direct or tacit expressions that will convey your attitudes.

### 3.2. Discover and Manage Patients’ Emotions

1. When possible, sit at eye level with the patient in a private environment that is conducive to open discussion, ideally without severe time constraints so patients can “think aloud” without feeling rushed.
2. To humanize the process, begin by saying a few things about yourself in relation to end-of-life decisions, e.g., “When I did this, I was amazed at how difficult it was to make good decisions. Now I would like to you cover the same bumpy ground.” Or “It is always challenging when I have helped other patients make similar decisions. But I have found by listening to each other and working together we have been able make decisions that helped them move forward confidently.”
3. Help the patient feel known as a person by asking, for example: “What are your three core values?” Or: “What three words best describe the person you want to be?” Or: “What are your three most important accomplishments in life?” Or: “What are your greatest hopes and fears?”
4. Ask open-ended versus yes/no questions, e.g., “What comes to mind when you think about dying?” vs. “Are you worried about dying?”
5. For critical items, repeat what you think patient said and ask if you interpreted it correctly. When possible, ask one or more follow-up questions to amplify your understanding.
6. Assess patients’ heath literacy and numeracy to make you aware of their ability to understand the words and numbers you will use by asking, for example: “what does it mean if I say that 15% of hospitalized patients who receive CPR survive to discharge?”
7. Rather than assuming that the patient is accurately informed, ask for a description of his/her current diagnosis, its prognosis, and the treatments that are possible. If there are factual misunderstandings, gently correct them, after stating that you realize how difficult it can be to understand medical language.
8. Pay careful attention to the nonverbal aspects of the patient’s responses. Does she or he listen to you, respond appropriately to various kinds of information, and ask questions that indicate an attempt to understand? When discordant with the content, nonverbal cues can also help with diagnosis. An example the “faint smile response” of parents with Munchausen’s-by-proxy when told that their child’s condition is serious.
9. After agreeing on the diagnosis and prognosis, discuss treatment options as follows:
  - “How likely is it that each of the options will help you reach your goal?”
  - “How much pain, discomfort, and cost are you willing to expend in order to accomplish your goal?”
  - “Which option would you like to choose first?” “What led you to this decision?”
  - “How do you think your significant other(s) will react to the decision, and how much do their reactions matter?”
  - “Are you committed to follow through on the decision?” If so, “What obstacles do you anticipate, and how do you plan to overcome them?” “If not, what alternatives are you considering?”
  - “How do you feel about this process?” “Have we failed to discuss anything else that is important to you?”
  - It is often necessary to repeat this discussion as the patient’s illness and its treatment progress, when “hope meets reality”.

### 3.3. Choose Materials That Personalize the Process and Outcome for Patients

Choice of material is also critical in efforts to structure the discussion in a patient-friendly format. Many patients feel disenfranchised by the medical system, in which providers wear uniforms and signs of status, speak a technical language that patients do not understand, and present binary options that appear dehumanizing and mechanistic, e.g., ACPR (Attempt Cardiopulmonary Resuscitation) vs. DNAR (Do Not Attempt Cardiopulmonary Resuscitation). Patients want the information they receive, and the choices they are asked to make, to be personalized rather than boilerplate, so that their plans can address their unique values, goals, and capacities. Doing so is the hallmark of personalized care [18,19].

Use of Conditional Medical Orders (CMO) personalizes patient engagement in shared decision making. CMOs allow patients to choose whether to accept an intervention “always, sometime, or never,” in keeping with their status at the time. This reassures patients that the care they undergo will accord closely with their condition and preferences when action is taken [20,21]. Using a CMO has the added advantage of increasing patient willingness to engage in ACP, and then to follow through on their choices.

Before he was diagnosed with Stage IV melanoma, 66-year-old John Smith’s Karnofsky Performance Status (KPS) was 90%, indicating normal activity with only minor signs or symptoms of disease. After undergoing first-line immunotherapy for his tumor, his cancer had progressed and his KPS declined to 70% indicating that he could care for himself but was unable to carry on normal activity or to do active work. He accepted the fact that further chemotherapy might extend his life a little, but at the cost of considerable discomfort. Because he wanted to optimize his physical and mental well-being during the time left to him, in discussion with his provider, John chose palliative care and did not want to be resuscitated if his illness caused his death. However, he did not want to lose the opportunity for meaningful contact with his family and friends if his heart stopped beating due to a potentially reversible cause such as a profound allergic reaction immediately after receiving a drug he had never taken before or a medical error. He therefore chose a DNAR-X.

**Figure 1:**
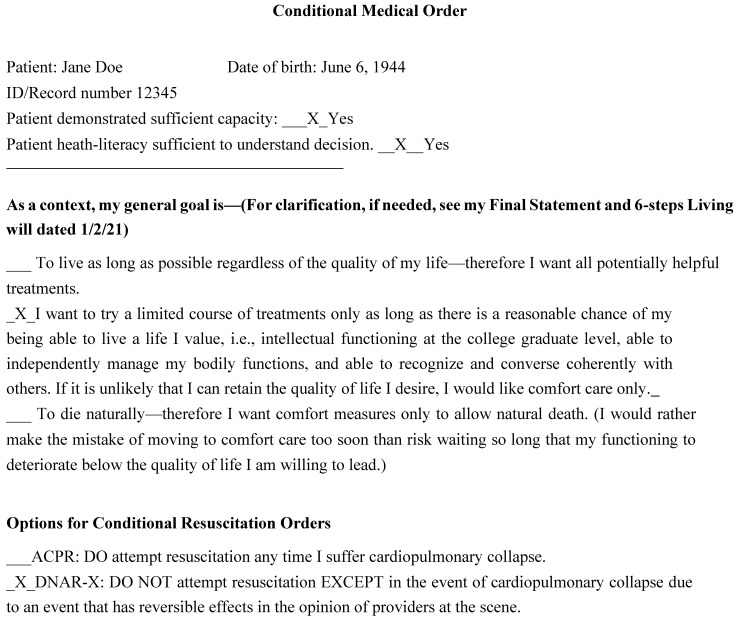

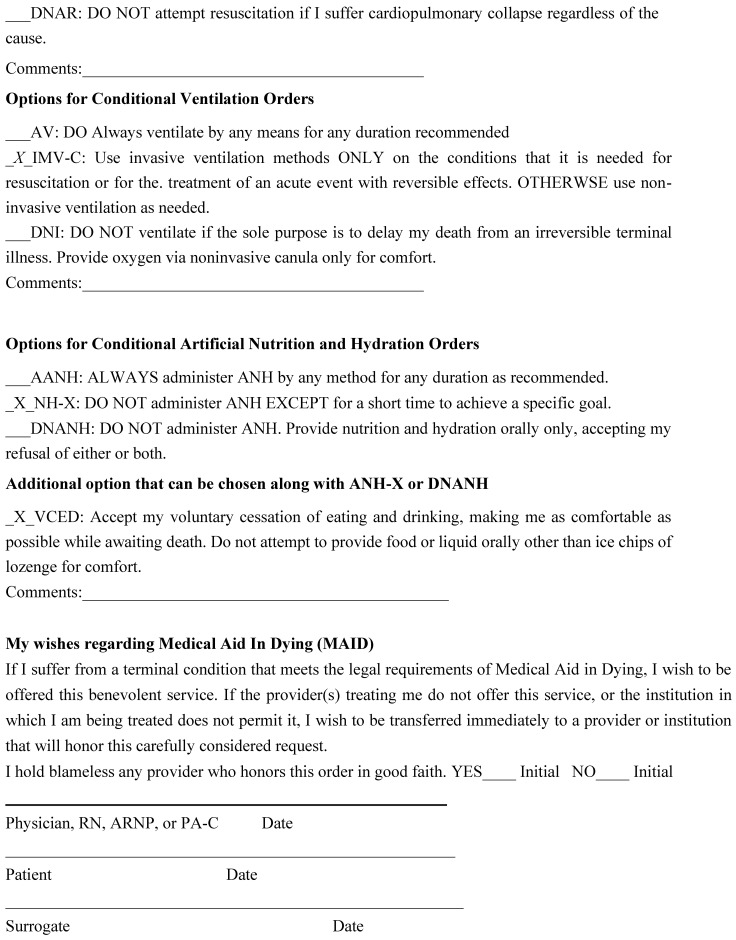
Sample Personalized Conditional Medical Order.

Figure 1 displays one way in which one patient customized the editable CMO in an effort to clarify her wishes in complex situations. Emotionally empathic sensitive motivational interviewing was necessary to help Ms. Doe clarify her thinking and formalize her preferences. When the document was complete, Ms. Doe observed: “Instead of just looking at the computer, this way I know that you will look at me when you decide whether I will live or die”.

## 4. Discussion

There may be some hesitation about efforts to address patients’ feelings about intervention rather than more efficiently focusing only on the facts. It definitely can be a more lengthy and complicated process. The World Health Organization acknowledged that “integrative care [is] more time-consuming and challenging than the current practice, [citing} human resource capacity as a barrier” [22]. However, facts alone do not drive patients’ behavior; emotions are often at often at least as powerful, if not more so. If time is spent making recommendations that patients will ignore, that time will be wasted, and the patients’ well-being will be compromised if they or their healthcare provider does not follow through later by readdressing the issue. Fortunately, counterbalancing the cost of more effective shared decision making is the fact that patients who participate in these discussions are more likely to choose less costly treatments [23,24]. For example, one study found that medical expenditures 6 and 3 months before death were significantly lower for patients who were able to discuss options such as palliative care with their providers [25]. Even more important is the likelihood that these discussions will lead to care that is more concordant with patients’ goals and values. There is great moral value in discussions that move patient care from a mechanistic style in which patients are “salvageable” to an organismic approach that recognizes patients’ unique perspectives [26].

When physician and nurse time is very tight, the providers can communicate basic diagnostic and prognostic information to social workers, chaplains, and other well-trained facilitators who can conduct the vital shared-decision-making sessions. These discussions can take place at the bedside, in a clinic or office, or remotely via phone or internet. As a caution, these discussions should be less focused on changing patients’ feelings than help them articulate and understand the likely outcome of acting on their emotions, particularly when the latter conflict with medical realities. Addressing patients’ emotions puts providers at the risk of compassionately sharing their distress, but this a worthwhile price to pay for the opportunity to more meaningfully connect with patients.

The relevance of advance care plans to critical care decisions is always a concern. It has been suggested that ACPs should disease-centered [27]. That would be ideal but it is impractical if, as recommended, planning takes place well in advance of the time they are needed because it is often impossible to anticipate precisely when that need will arise. The CMO comes closest to meeting this criteria by stipulating the conditions under which interventions will be accepted contingent on their effects in the trajectory of any illness. CMOs can be personalized when the details are known.

As a final consideration, once it is understood that emotions profoundly influence what patients learn from providers and how they act on the information they are given, it becomes ethically necessary to address patients’ emotions during shared decision-making. Failing to do so puts patients at greater risk of making poor decisions or failing to follow through on wise choices and puts providers at risk of offering substandard care.

## 5. Conclusions

Palliative care discussions are an optimal time for ACP. It is easy for providers to ascribe the sparce use of ACP to patients’ lack of motivation and understanding because many people do wish to avoid thinking about illness and dying. Yet, another key factor is providers’ reluctance to discuss death, which many consider a failure rather than an inevitability, and providers’ preference to deal with facts rather than with emotions. Because providers are trained to focus on the science of medicine, it is understandable that many are not equally attentive to the process of emotionally sensitive treatment planning. Attending adequately to emotions requires providers to become aware of and manage their own feelings, because these often profoundly influence patients’ motivation to meaningfully engage in the process. In addition to careful attention to emotions, use of motivational interviewing techniques and choice of personalized and patient-friendly materials like the CMO can help ACP reach its vast potential to improve end-of-life care.
